# Comparison of Gating Properties and Use-Dependent Block of Na_v_1.5 and Na_v_1.7 Channels by Anti-Arrhythmics Mexiletine and Lidocaine

**DOI:** 10.1371/journal.pone.0128653

**Published:** 2015-06-11

**Authors:** Ying Wang, Jianxun Mi, Ka Lu, Yanxin Lu, KeWei Wang

**Affiliations:** 1 Biomedical Research Institute, Shenzhen Peking University-Hong Kong University of Science and Technology Medical Center, Shenzhen 518036, China; 2 Key Laboratory of Computational Intelligence, College of Computer Science and Technology, Chongqing University of Posts and Telecommunications, Chongqing 400065, China; 3 Department of Molecular and Cellular Pharmacology, State Key Laboratory of Natural and Biomimetic Drugs, Peking University School of Pharmaceutical Sciences, Beijing 100191, China; 4 Department of Pharmacology, Qingdao University School of Pharmacy, Qingdao 266021, China; University at Buffalo, UNITED STATES

## Abstract

Mexiletine and lidocaine are widely used class IB anti-arrhythmic drugs that are considered to act by blocking voltage-gated open sodium currents for treatment of ventricular arrhythmias and relief of pain. To gain mechanistic insights into action of anti-arrhythmics, we characterized biophysical properties of Na_v_1.5 and Na_v_1.7 channels stably expressed in HEK293 cells and compared their use-dependent block in response to mexiletine and lidocaine using whole-cell patch clamp recordings. While the voltage-dependent activation of Na_v_1.5 or Na_v_1.7 was not affected by mexiletine and lidocaine, the steady-state fast and slow inactivation of Na_v_1.5 and Na_v_1.7 were significantly shifted to hyperpolarized direction by either mexiletine or lidocaine in dose-dependent manner. Both mexiletine and lidocaine enhanced the slow component of closed-state inactivation, with mexiletine exerting stronger inhibition on either Na_v_1.5 or Na_v_1.7. The recovery from inactivation of Na_v_1.5 or Na_v_1.7 was significantly prolonged by mexiletine compared to lidocaine. Furthermore, mexiletine displayed a pronounced and prominent use-dependent inhibition of Na_v_1.5 than lidocaine, but not Na_v_1.7 channels. Taken together, our findings demonstrate differential responses to blockade by mexiletine and lidocaine that preferentially affect the gating of Na_v_1.5, as compared to Na_v_1.7; and mexiletine exhibits stronger use-dependent block of Na_v_1.5. The differential gating properties of Na_v_1.5 and Na_v_1.7 in response to mexiletine and lidocaine may help explain the drug effectiveness and advance in new designs of safe and specific sodium channel blockers for treatment of cardiac arrhythmia or pain.

## Introduction

Voltage-gated sodium channels (VGSCs) play an important role in the initiation and propagation of action potential in excitable cells. In principle, they are over 50% identical in amino acid sequence, but exhibit distinct gating properties and pharmacology between the tetrodotoxin-resistant (TTX-R) and tetrodotoxin-sensitive (TTX-S) sodium channels [1]. TTX-R Na_v_1.5 channel is blocked by micromolar concentration of TTX, while TTX-S Na_v_1.7 channel is more sensitive to TTX block at nanomolar range. Na_v_1.5 channel is the major voltage-gated channel isoform in adult heart, playing a key role in generating rhythm of heartbeat. Na_v_1.7 channel is expressed at high levels in pain-sensing dorsal root ganglia (DRG) neurons and sympathetic ganglia neurons, involving in acute and chronic pain [2]. Despite the fact that both Na_v_1.5 and Na_v_1.7 are inhibited by a variety of chemical compounds, the isoform-specific blockers are lacking.

The fundamental function of voltage-gated sodium channels is characterized by their gating properties that include channel activation, inactivation, and recovery from inactivation (also called ‘reactivation’) [3, 4]. In particular, inactivation (the non-conductive state) taking place during sustained depolarization is dynamic and essential for modulation of membrane excitability. Inactivation has different processes including open-state inactivation (OSI) that occurs from the open state at strongly depolarized potentials, and closed-state inactivation (CSI) that happens from pre-open closed states at hyperpolarized or modestly depolarized potentials [5]. Inactivation is featured of fast inactivation (complete within a few milliseconds) and slow inactivation (ranging from ~100 ms to several minutes) [3, 6, 7]. Inactivation gating is an intrinsic self-regulatory process requisite for regulating occurrence and shape of action potentials and setting up firing patterns in excitable tissues [5]. During repetitive stimulation or a train of depolarizing pulses, the number of sodium channels available to open reduces since they gradually accumulate in the inactivated state, causing a use-dependent or frequency-dependent inhibition [8]. This inhibition can be considered as a result of different gating properties responding to the repetitive stimulation.

Mexiletine and lidocaine belong to class IB anti-arrhythmic drugs that are widely used for treatment of life-threatening ventricular arrhythmias. Mexiletine is an orally active congener of lidocaine. In clinical investigations and practice, mexiletine has been shown to be more effective for gene-specific therapy for patients with long-QT syndrome type 3 (LQT3) caused by *SCN5A* gain-of-function mutations that dramatically affect Na_v_1.5 channel gating [9, 10]. It has been suggested that gating properties of voltage-gated sodium channels can be used as a basis to predict drug response for effective treatment of ventricular tachycardia [11]. As nonselective inhibitors of sodium channels, mexiletine also presents beneficial effects on pain through inhibition of Na_v_1.7 channels [12–14]. Lidocaine is widely used as local anesthetic (LA) for treatment of neuropathic pain at higher doses. It also can selectively target cardiac sodium channels for treatment of arrhythmias at micromolar concentrations with few side effects on muscle or neuronal subtypes. A mathematical model of atrial and ventricular cardiomyocytes predicts that lidocaine is more AF (atrial fibrillation)-selective and less proarrhythmic, thus ideal for the prevention and treatment of AF in clinical medicine [15]. Although most patients tolerate lidocaine well, certain people might experience severe side effects [16]. Specifically, lidocaine results in slow-down of heartbeat, thus leading to cardiac arrest [17]. Besides mexiletine or lidocaine, several compounds such as ranolazine, benzocaine and flecainide have also been shown to block sodium channel isoforms [18–20]. However, little is understood about how sodium channel blockers including mexiletine and lidocaine differentially affect the gating of different voltage-gated sodium isoforms [21]. Establishing a detailed pharmacological basis of Na_v_1.5 and Na_v_1.7 channels can provide valuable insight into the drug’s action for developing safe and effective therapies to treat arrhythmia and to alleviate pain.

In this study, we investigated gating properties of Na_v_1.5 and Na_v_1.7 channels and examined effects of mexiletine and lidocaine on channel activation, inactivation and recovery from inactivation, and compared use-dependent block of Na_v_1.5 and Na_v_1.7 channels. Our findings demonstrate that anti-arrhythmic drugs mexiletine and lidocaine preferentially affect the gating of Na_v_1.5 as compared to Na_v_1.7, whereas mexiletine exhibits a stronger use-dependent block on Na_v_1.5.

## Materials and Methods

### Cell culture

HEK293 cells stably expressing human homolog of human Na_v_1.5 (SCN5A, NM_198056.2) and human Na_v_1.7 (SCN9A, NM_002977.1) channels were maintained under standard tissue culture conditions (5% CO2; 37°C) in Dulbecco’s modified Eagle’s medium (Invitrogen) and F-12 medium (Invitrogen) mixture at 1:1 supplemented with 10% fetal bovine serum (Hyclone) under selection of antibiotic G418 (Genecitin; Invitrogen) with a concentration of 500 μg/mL. Cells were passaged every three days with 0.25% Trypsin-EDTA (Invitrogen). Whole-cell recordings were performed 18–24 h after plating at room temperature.

### Electrophysiology

The pipette solution contained (in mM): 140 CsF, 10 NaCl, 1 EGTA, and 10 HEPES; pH 7.3 with CsOH. The bath solution for recording contained (in mM): 140 NaCl, 3 KCl, 10 HEPES, 1 MgCl_2_, 1 CaCl_2_; pH 7.3 with NaOH. Fluoride was contained in the intracellular solution to perform tests with extended voltage-clamp protocols and examine the drug’s interaction with slow inactivation. Although it has been reported that fluoride can shift the voltage-dependent curves about -15 to -20 mV [14, 22], our data were not corrected for this. Mexiletine and lidocaine (Sigma) were diluted in bath solution to the desired concentration from a stock solution (10 mM in distilled water stored in dark at 4°C).

Whole-cell patch-clamp recordings were conducted at room temperature (22–25°C) using an EPC-10 USB amplifier. Data were acquired using the Patchmaster program (version 2.30; HEKA Electronic). Electrodes (1.8–2.5 MΩ) were fabricated from 1.5 mm Sutter Instrument BF150-75-10 borosilicate glasses with filament using a DMZ-Universal puller (Zeitz-Instruments, Germany). The average access resistance was 2–4 MΩ and the seal resistance during recordings was at least 600 MΩ. Cells were discarded if the access resistance was over 4 MΩ or the seal resistance during recording was below 600 MΩ. Series resistance compensation was always set at the highest level for each cell (88–91%). Capacity transients were cancelled via the computer-controlled circuitry of patch-clamp amplifier. Leak subtraction was conducted by using P/4 procedure after the test pulse. Currents were filtered at 2.9 kHz of Bessel filter and sampled at 10 kHz. Recordings were generally initiated approximately 5 min after establishment of whole-cell configuration. The liquid junction potential was not corrected.

### Data analysis

Data were analyzed using the IgorPro (Version 6.02; Wavemetrics, Inc), Origin (Version 7.5; Microcal Software) and MATLAB (The MathWorks, Inc.) software programs. Statistical significance was determined by *p < 0.05, **p < 0.01; and ***p < 0.001 using unpaired t-test or ANOVA. Whenever stated with using ANOVA, we also performed multiple comparison tests for the means (Dunnett difference criterion) to assign an indication of significance between control and drug groups. In some cases, we used Bonferroni multiple comparison tests between drug groups and specified them in the text. If there was no significance between samples, we provided the actual *p*-values. Pooled data are presented as mean ± s.e.m. and error bars in the figures represent SEs.

Voltage-dependent activation was measured by applying a series of step depolarization to indicated voltages (-80 to +40 mV in 5 mV increments with 5 s interval for 50 ms) from a holding potential of -120 mV. Peak current at each voltage was measured and corresponding conductance (G) was calculated by using the equation: G = I / (V - V_rev_), where V is the voltage test, and V_rev_ was calculated by linear extrapolation of peak currents with depolarization potentials from 10 to 40 mV. Normalized G was then plotted versus voltage and activation curves were obtained by Boltzmann fitting [23]: G/G_max_ = 1/(1+exp((V_1/2_-V)/*k*)), where G_max_ is the maximal sodium conductance, and V_1/2_ is the potential of half-maximal activation and *k* is the slope factor. The decay time constants reflecting inactivation kinetics were estimated from one exponential fitting of the decay phase of currents elicited by the 50 ms pulses to indicated voltages (from -40 mV to +40 mV).

Steady-state fast inactivation was tested by measuring the fraction of current available for activation by a 50 ms step depolarization to -20 mV after a 750 ms conditioning pulse ranging from -120 mV to +20 mV in 10 mV step increments, from a holding potential of -120 mV. Normalized residual current was plotted versus the voltage of conditioning pulse. Steady-state fast inactivation curves were fitted with Boltzmann function again: I/I_max_ = 1/(1+exp((V_1/2_-V)/*k*)) [23].

Steady-state slow inactivation was examined with 10 s prepulse at voltages ranging from -120 mV to +20 mV in 10 mV step increments followed by a 100 ms hyperpolarization gap at -120 mV to remove fast inactivation. The remaining available channels were evoked by a 50 ms test pulse to -20 mV. Normalized residual current was plotted versus the voltage of conditioning pulse. Steady-state slow inactivation curves were fitted with Boltzmann function as described for fast inactivation [23].

Development of closed-state inactivation was measured by protocols in which holding potential was set at -120 mV, and conditioning pulse was set at -80 mV [24]. Cells were depolarized to conditioning pulse for various duration (0.1 ms–9 s) before test pulse at -20 mV. The fraction of channels available following conditioning pulse was plotted versus time. Closed-state inactivation curves were fitted by two-exponential function.

Recovery from inactivation was measured by using paired-pulse protocols in which cells were held at -120 mV before first pulse at -20 mV for 50 ms. Channels were then allowed to recover using a hyperpolarizing pulse (equal to the holding potential) for a various duration before a second test pulse at -20 mV for 50 ms. Fraction of channel recovery was determined by normalizing the current elicited from the second test pulse to the first conditioning pulse, and then plotted versus the recovery time (0.1 ms–1.6 s). Curves were obtained by two-exponential fitting.

Use/Frequency-dependent blockade of channel was measured by applying repetitive pulses at different frequencies (1 Hz, 5 Hz and 10 Hz). The individual pulses at 1 Hz and 5 Hz were applied for 50 ms to -20 mV, whereas pulses at 10 Hz were 25 ms in duration. The peak current evoked by each pulse was normalized to the current induced by first pulse and plotted against pulse numbers.

## Results

### Functional expression of Na_v_1.5 and Na_v_1.7 channels and lack of effects of mexiletine and lidocaine on voltage-dependent activation

**Fig 1:**
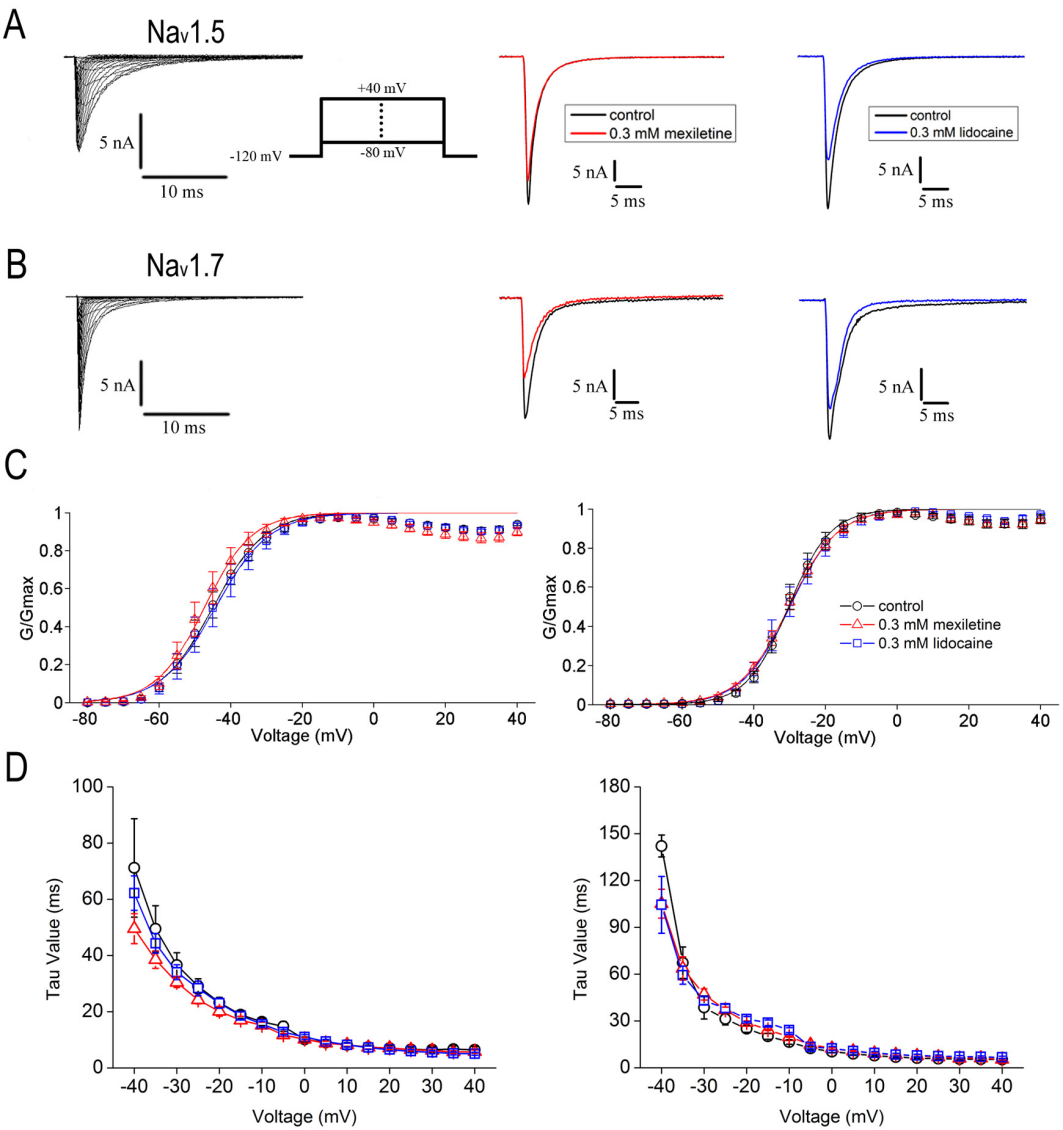
Effects of mexiletine and lidocaine on voltage-dependent activation of Na_v_1.5 and Na_v_1.7 channels. A, B, Representative activation traces were recorded in HEK293 cells stably expressing either Na_v_1.5 (A, left panel) or Na_v_1.7 (B, left panel) channels. In the presence of 0.3 mM mexiletine (middle panel) or lidocaine (right panel), the peak currents of both channels evoked by 50 ms pulses to -20 mV were inhibited. The protocol of voltage-dependent activation is shown in A inset. C, Normalized conductance versus voltage was plotted for Na_v_1.5 (left panel) and Na_v_1.7 (right panel) channels. Curves were fitted by Boltzmann function: G/Gmax = 1-1/ (1 + exp ((V-V_1/2_)/*k*)). The V_1/2_ and slope factor (*k*) of Na_v_1.5 and Na_v_1.7 channels are listed in Table 1. Mexiletine and lidocaine caused no significant shift of V_1/2_ and slope factor for both Na_v_1.5 and Na_v_1.7 channels. D, Effects of mexiletine and lidocaine on inactivation kinetics. The inactivation decay time constants were estimated with one exponential fit from the decay of current elicited by a 50 ms pulse to indicated voltages. Mexiletine or lidocaine slightly but no significantly accelerated the decay phase of Na_v_1.5 at -40 mV and -35 mV (left panel), whereas slightly accelerated decay of Na_v_1.7 inactivation was only found at -40 mV (right panel).

To examine the effects of mexiletine and lidocaine on channel gating properties, we started recording of voltage-dependent activation of Na_v_1.5 and Na_v_1.7 channels stably expressed in HEK293 cells. The activation of Na_v_1.5 or Na_v_1.7 channels induces inward Na^+^ currents. As shown in Fig 1, a family of depolarizing steps by holding at -120 mV and then applying 50 ms depolarizing pulses to potentials from -80 mV to +40 mV in 5 mV increment (Fig 1A, inset), elicited robust instantaneous voltage-dependent activation and rapid inactivation of whole-cell Na_v_1.5 or Na_v_1.7 currents (Fig 1A and 1B). Bath application of 0.3 mM mexiletine or lidocaine resulted in reduction of Na_v_1.5 or Na_v_1.7 currents (Fig 1A and 1B).

The I-V curves were transformed to G-V curves and fitted with Boltzmann function for generation of the midpoint voltage (V_1/2_) and the slope factor (*k*) (see Fig 1C and Table 1). The V_1/2_ values for Na_v_1.5 were not significantly shifted in the presence of mexiletine at 0.3 mM (V_1/2_ = -47.0 ± 1.6 mV, n = 10) and 1.0 mM (V_1/2_ = -47.0 ± 1.1 mV, n = 10) (*p* = 0.5505, one-way ANOVA, F = 0.61) or lidocaine at 0.3 mM (V_1/2_ = -40.1 ± 1.9 mV, n = 11) and 1.0 mM (V_1/2_ = -41.3 ± 0.9 mV, n = 13) (*p* = 0.1379, one-way ANOVA, F = 2.08), as compared with the blank control without drugs (V_1/2_ = -44.7 ± 1.8 mV, n = 21). Similarly, the V_1/2_ values for Na_v_1.7 remained almost unchanged for mexiletine (V_1/2_ = -30.3 ± 1.1 mV, n = 14 for 0.3 mM and V_1/2_ = -29.9 ± 0.9 mV, n = 18 for 1 mM; *p* = 0.0729, one-way ANOVA, F = 2.81) and lidocaine (V_1/2_ = -29.9 ± 1.9 mV, n = 13 for 0.3 mM and V_1/2_ = -29.4 ± 1.2 mV, n = 9 for 1 mM; *p* = 0.0737, one-way ANOVA, F = 2.88), as compared with the control (V_1/2_ = -30.4 ± 1.2 mV, n = 8). Increasing mexiletine or lidocaine concentration (1.0 mM) had no effects on activation in both Na_v_1.5 and Na_v_1.7 channels (Table 1).

**Table 1:** Parameters obtained from voltage-dependent activation curve fittings with Boltzmann function.

	Na_v_1.5		Na_v_1.7	
	V_1/2_ (mV)	k	V_1/2_ (mV)	k
**control**	-44.7 ± 1.8 (n = 21)	5.2 ± 0.5	-30.4 ± 1.2 (n = 8)	4.0 ± 0.6
**mexiletine 0.3 mM**	-47.0 ± 1.6 (n = 10)	5.3 ± 0.4	-30.3 ± 1.1 (n = 14)	6.1 ± 0.4
**mexiletine 1.0 mM**	-46.9 ± 1.1 (n = 10)	5.8 ± 0.6	-29.9 ± 0.9 (n = 18)	6.5 ± 0.2
**lidocaine 0.3 mM**	-40.1 ± 1.9 (n = 11)	5.4 ± 0.7	-29.9 ± 1.9 (n = 13)	5.8 ± 0.4
**lidocaine 1.0 mM**	-41.3 ± 0.9 (n = 13)	5.6 ± 0.3	-29.4 ± 1.2 (n = 9)	6.4 ± 0.5

We also measured the decay time constants of inactivation after channel activation. The decay time constants were estimated with one-exponential fitting from the decay current elicited by a 50 ms pulse to indicated voltages (Fig 1D). Mexiletine or lidocaine at 0.3 mM slightly but not significantly accelerated the decay phase of Na_v_1.5 at -40 mV (*p* = 0.5109, one-way ANOVA, F = 0.70) and -35 mV (*p* = 0.4757, one-way ANOVA, F = 0.78), whereas slightly accelerated decay of Na_v_1.7 inactivation was only found at -40 mV (Fig 1D, *p*<0.05, one-way ANOVA, F = 4.78).

### A hyperpolarizing shift of steady-state fast inactivation of Na_v_1.5 and Na_v_1.7 channels in the presence of mexiletine or lidocaine

Voltage-gated sodium channels inactivate rapidly within millisecond range and inactivate slowly ranging from about 100 ms to several minutes after opening in response to depolarization [6]. This inactivation process is required for repetitive firing of action potential and for modulation of excitability in excitable cells. When resting membrane potential moves towards more depolarized voltages, channels in non-conducting inactivated state are accumulated and fewer channels become available.

**Fig 2:**
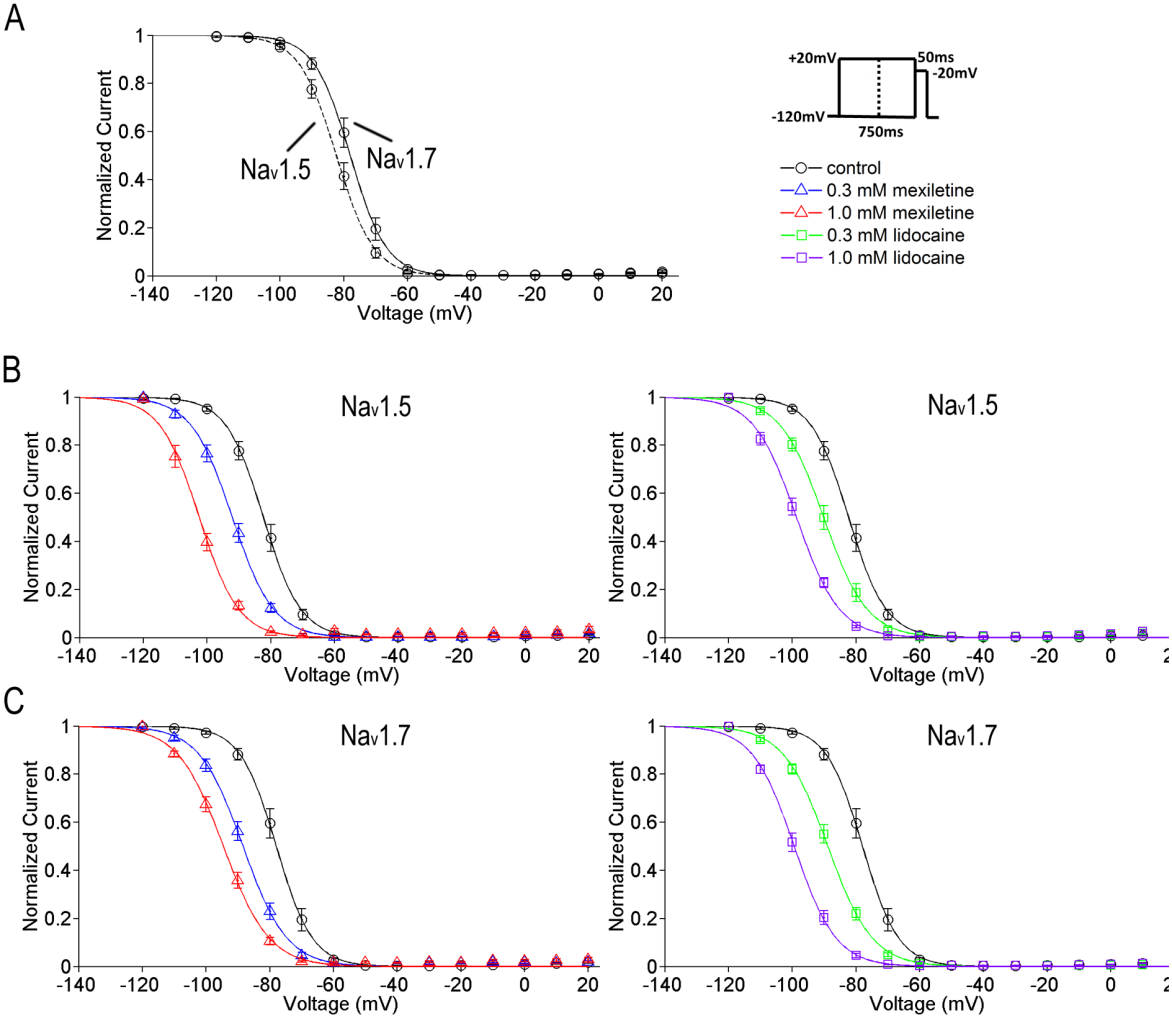
A hyperpolarizing shift of steady-state fast inactivation of Na_v_1.5 and Na_v_1.7 channels by mexiletine and lidocaine. Steady-state fast inactivation curves of Na_v_1.5 and Na_v_1.7 channels were obtained by Boltzmann fitting: I/Imax = 1/ (1 + exp ((V-V_1/2_)/*k*)). The V_1/2_ and slope factor (*k*) of Na_v_1.5 and Na_v_1.7 channels are listed in Table 2. A. The steady-state channel availability between Na_v_1.5 and Na_v_1.7 has a 5.5-mV difference (Na_v_1.5: V_1/2_ = -83.5 ± 1.4, n = 23; Na_v_1.7: V_1/2_ = 78.0 ± 1.4, n = 8; p<0.05, unpaired t-test,). The protocol is shown in right panel. B and C, the V_1/2_ values of both channels were shifted to the hyperpolarized direction significantly at 0.3 mM and 1 mM of mexiletine or lidocaine. The shift displayed a concentration-dependent effect. Both drugs caused the curves less steep compared to drug-free condition. See Table 2 for all the time constants.

To investigate effects of mexiletine and lidocaine on channel fast inactivation, we applied a constant 750 ms conditioning pulse (as shown in Fig 2A, right panel) and detected about 5.5-mV difference in voltage dependence of sodium current availability for the two sodium channel isoforms in blank control condition (Na_v_1.5: V_1/2_ = -83.5 ± 1.4 mV, n = 23; Na_v_1.7: V_1/2_ = -78.0 ± 1.4 mV, n = 8; *p*<0.05, unpaired t-test, Fig 2A). When either mexiletine or lidocaine applied, steady-state fast inactivation of both Na_v_1.5 and Na_v_1.7 channels was significantly shifted to the negative hyperpolarized direction in a dose-dependent manner. Mexiletine at 0.3 mM and 1.0 mM caused a shift of V_1/2_ for Na_v_1.5 to the negative direction by 8.8 mV (n = 12) and 20.3 mV (n = 11), respectively, as compared with the control (*p*<0.001, one-way ANOVA, F = 39.59). Lidocaine at 0.3 mM and 1.0 mM shifted the V_1/2_ for Na_v_1.5 to the left by 6.6 mV (n = 12) and 16.7 mV (n = 15), respectively (*p*<0.001, one-way ANOVA, F = 38.68; Fig 2B and Table 2). For Na_v_1.7, mexiletine at 0.3 mM and 1.0 mM shifted the V_1/2_ to the left by 10.7 mV (n = 13) and 17.2 mV (n = 12), respectively (*p*<0.001, one-way ANOVA, F = 45.87). Lidocaine at 0.3 mM and 1.0 mM shifted the V_1/2_ for Na_v_1.7 to the left by 10.9 mV (n = 8) and 23.6 mV (n = 7), respectively (*p*<0.001, one-way ANOVA, F = 79.56; Fig 2C and Table 2). Both drugs made the fast inactivation curves less steep in Na_v_1.5 (*p*<0.001, one-way ANOVA, F = 32.09) and Na_v_1.7 channels (*p*<0.001, one-way ANOVA, F = 25.36).

**Table 2:** Parameters obtained from steady-state fast inactivation curve fittings with Boltzmann Function.

	Na_v_1.5		Na_v_1.7	
	V_1/2_ (mV)	k	V_1/2_ (mV)	k
**control**	-83.5 ± 1.4 (n = 23)	4.6 ± 0.1	-78.0 ± 1.4 (n = 8)	5.0 ± 0.2
**mexiletine 0.3 mM**	-92.3 ± 1.2 (n = 12) ***	6.0 ± 0.2***	-88.7 ± 1.1 (n = 13) ***	6.3 ± 0.3***
**mexiletine 1.0 mM**	-103.8 ± 1.4 (n = 11) ***	7.0 ± 0.2***	-95.2 ± 1.1 (n = 12) ***	6.7 ± 0.2***
**lidocaine 0.3 mM**	-90.1 ± 1.3 (n = 12) ***	6.2 ± 0.2***	-88.9 ± 1.1 (n = 8) ***	6.7 ± 0.2***
**lidocaine 1.0 mM**	-100.2 ± 1.3 (n = 15) ***	6.9 ± 0.2***	-101.6 ± 1.4 (n = 7) ***	7.0 ± 0.2***

Note: Asterisk indicates values derived from fitting the Boltzmann function that differ from the corresponding values in control (drug-free) condition.

***P < 0.001, one-way ANOVA.

**Fig 3:**
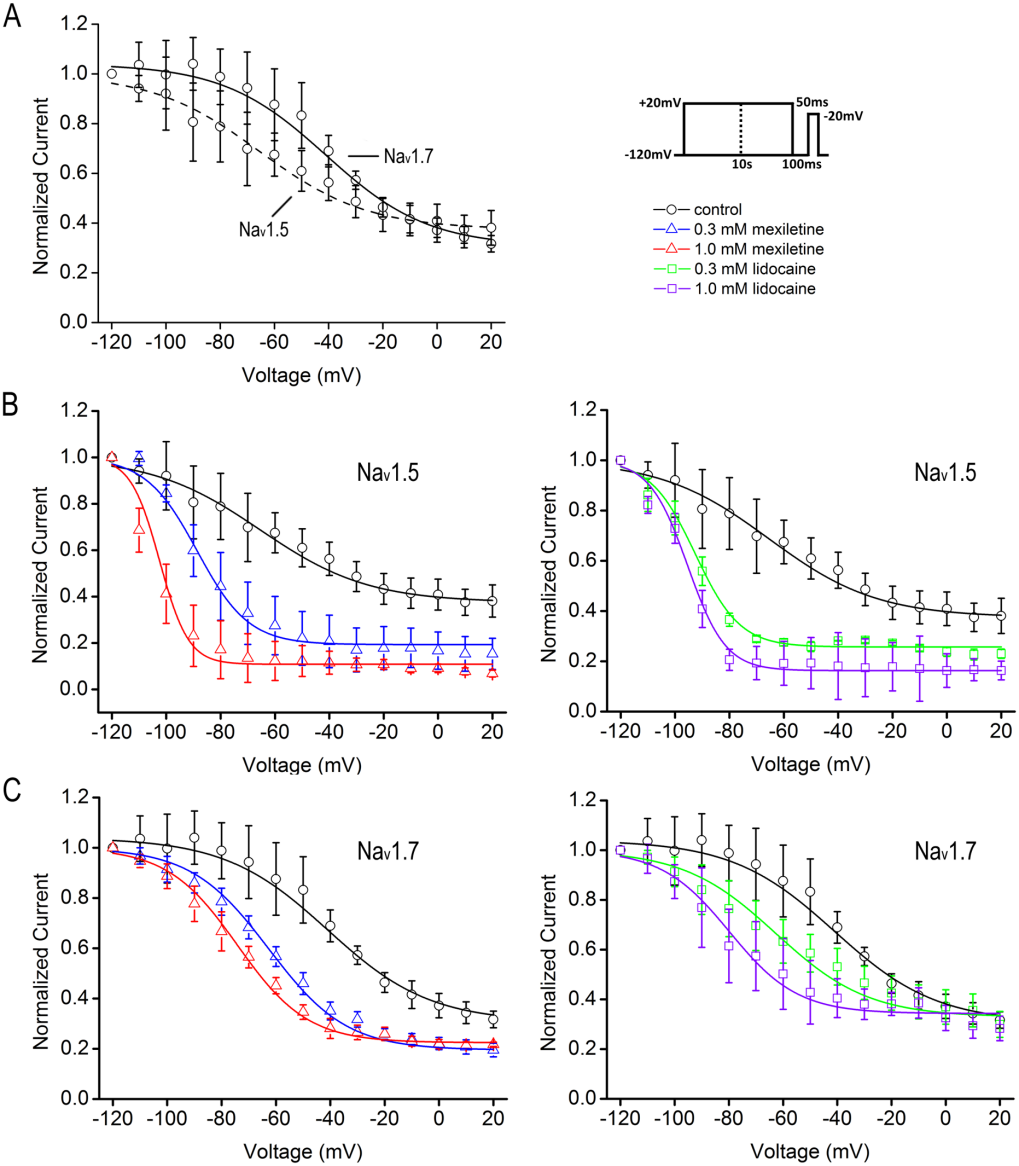
A hyperpolarizing shift of steady-state slow inactivation of Na_v_1.5 and Na_v_1.7 channels by mexiletine and lidocaine. Steady-state slow inactivation curves of Na_v_1.5 and Na_v_1.7 channels were obtained by Boltzmann fitting: I/Imax = 1/ (1 + exp ((V-V_1/2_)/*k*)). The V_1/2_ and slope factor (*k*) of Na_v_1.5 and Na_v_1.7 channels are listed in Table 3. A. The midpoint of Na_v_1.5 is more hyperpolarized than that of Na_v_1.7 (Na_v_1.5: V_1/2_ = -65.4 ± 11.9 mV, n = 5; Na_v_1.7: V_1/2_ = -41.0 ± 3.4 mV, n = 5; p<0.001, unpaired t-test). The protocol is shown in right panel. B and C, the V_1/2_ values of both channels were shifted to the hyperpolarized direction significantly at 0.3 mM and 1 mM of mexiletine or lidocaine. The shift displayed a concentration-dependent effect. The steepness factor is similar as the control for Na_v_1.7, while for Na_v_1.5 the curves become steeper in the presence of mexiletine or lidocaine. See Table 3 for all the time constants.

Slow inactivation, which determines the propensity to generate repetitive firing and the extent of action potential backpropagation, is functionally distinct from fast inactivation [6]. We used the protocol of a constant 10 s conditioning pulse and measured the fraction of available currents. We detected a prominent 24.4 mV difference between two isoforms for steady-state slow inactivation in control condition (Na_v_1.5: V_1/2_ = -65.4 ± 11.9 mV, n = 5; Na_v_1.7: V_1/2_ = -41.0 ± 3.4 mV, n = 5; *p*<0.001, unpaired t-test). As similar with steady-state fast inactivation, the slow component displayed a negative hyperpolarized shift under the effects of mexiletine or lidocaine in a dose-dependent manner. Mexiletine at 0.3 mM and 1.0 mM caused a shift of V_1/2_ for Na_v_1.5 to the negative direction by 23.2 mV (n = 5) and 37.0 mV (n = 6), respectively, as compared with the control (*p*<0.05, one-way ANOVA, F = 5.76). Lidocaine at 0.3 mM and 1.0 mM shifted the V_1/2_ for Na_v_1.5 to the left by 27.2 mV (n = 7) and 30.0 mV (n = 5), respectively (*p*<0.001, one-way ANOVA, F = 22.87, Fig 3B and Table 3). For Na_v_1.7, mexiletine at 0.3 mM and 1.0 mM shifted the V_1/2_ to the left by 20.7 mV (n = 5) and 33.1 mV (n = 5), respectively (*p*<0.01, one-way ANOVA, F = 9.77). Lidocaine at 0.3 mM and 1.0 mM shifted the V_1/2_ for Na_v_1.7 to the left by 21.6 mV (n = 5) and 38.7 mV (n = 6), respectively (*p*<0.05, one-way ANOVA, F = 6.54; Fig 3C and Table 3). The steepness factor is similar to the control for Na_v_1.7 (*p* = 0.3482, one-way ANOVA, F = 1.19), whereas for Na_v_1.5 the curves became steeper in the presence of mexiletine or lidocaine (*p*<0.01, one-way ANOVA, F = 5.42).

**Table 3:** Parameters obtained from steady-state slow inactivation curve fittings with Boltzmann Function.

	Na_v_1.5		Na_v_1.7	
	V_1/2_ (mV)	k	V_1/2_ (mV)	k
**control**	-65.4 ± 11.9 (n = 5)	27.0 ± 5.7	-41.0 ± 3.4 (n = 5)	18.9 ± 3.0
**mexiletine 0.3 mM**	-88.6 ± 8.5 (n = 5) *	9.5 ± 6.9**	-61.7 ± 6.1 (n = 5) **	14.3± 1.5
**mexiletine 1.0 mM**	-102.4 ± 9.0 (n = 6) *	8.5 ± 1.7**	-74.1 ± 9.2 (n = 5) **	12.9± 3.3
**lidocaine 0.3 mM**	-92.6 ± 3.3 (n = 7) ***	10.8± 1.8**	-62.6 ± 12.5 (n = 5) *	17.0 ± 5.8
**lidocaine 1.0 mM**	-95.4 ± 3.0 (n = 5) ***	7.1 ± 1.5**	-79.7 ± 8.7 (n = 6) *	12.6 ± 5.1

Note: Asterisk indicates values derived from fitting the Boltzmann function that differ from the corresponding values in control (drug-free) condition.

*p < 0.05

**p < 0.01

***p < 0.001, one-way ANOVA

These results showed that both mexiletine and lidocaine significantly caused the shift of voltage-dependent inactivation of Na_v_1.5 or Na_v_1.7 to the hyperpolarized direction, reflecting a shift in equilibrium from resting to inactivated states, and thus providing a basis for suppressing repetitive firing.

### Facilitation of closed-state inactivation by mexiletine and lidocaine

**Fig 4:**
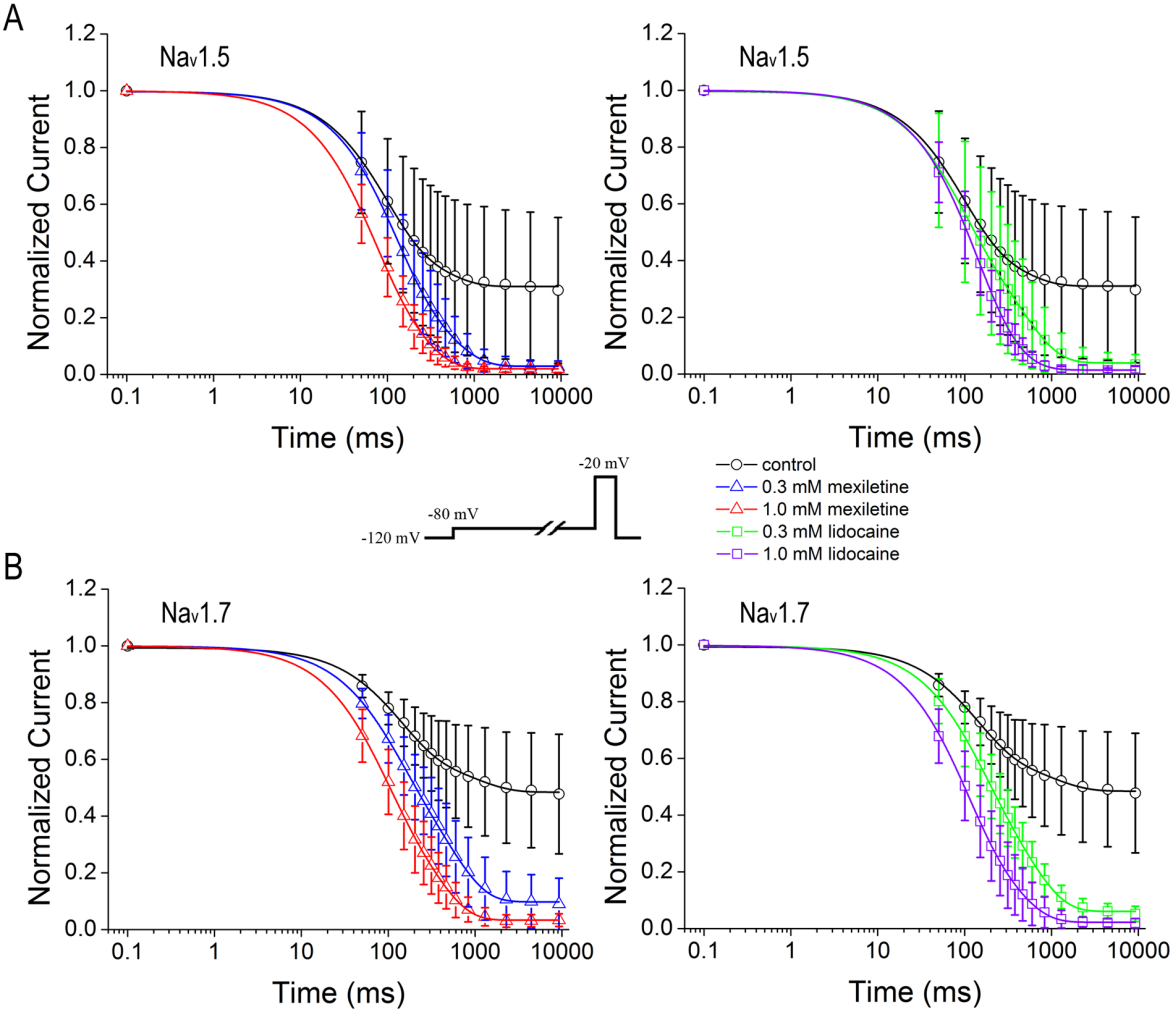
Enhanced closed-state inactivation of Na_v_1.5 and Na_v_1.7 channels by mexiletine and lidocaine. The closed-state inactivation curves were best fitted with two-exponential function. Both mexiletine and lidocaine accelerated the development of closed-state inactivation in Na_v_1.5 (A) and Na_v_1.7 (B) channels. The fast (τ_1_) and slow (τ_2_) constants of Na_v_1.5 and Na_v_1.7 channels are in the same range in control condition. All the τ values and the corresponding fraction in each condition are summarized in Table 4. Note that both mexiletine and lidocaine caused a significant reduction of slow τ_2_ values in dose-dependent manner, leading to speedy inactivation of either Na_v_1.5 or Na_v_1.7.

Inactivation can occur from pre-open closed states (closed-state inactivation, CSI) at modestly depolarized and hyperpolarized membrane potentials. Voltage-gated sodium channels utilize CSI to regulate firing frequency. To test the effects of mexiletine and lidocaine on CSI, we generated a depolarization protocol that contains a conditioning pulse at -80 mV with variable intervals from 0.1 ms to 10 s and a subsequent test pulse at -20 mV to measure the availability of Na_v_1.5 or Na_v_1.7 currents (Fig 4, inset). The onset of CSI was best fitted with the sum of two exponential, τ_1_ and τ_2_, representing time constants of fast and slow inactivation, respectively (Table 4). We found that the fast inactivation constants, τ_1_ values for both channels, were in the same range with Na_v_1.5 at 121.7 ± 10.3 ms and Na_v_1.7 at 126.7 ± 16.8 ms (*p* = 0.8035, unpaired t-test). In the presence of 0.3 or 1.0 mM lidocaine, there was little decrease for τ_1_ values of both Na_v_1.5 and Na_v_1.7, as compared with control (*p* = 0.7107, F = 0.36 for Na_v_1.5; and *p* = 0.6091, F = 0.5077 for Na_v_1.7, one-way ANOVA, Fig 4 and Table 4). In contrast, mexiletine (1 mM) caused a faster CSI with about 47% decrease of τ_1_ value for Na_v_1.5 and about 41% decrease of τ_1_ value for Na_v_1.7. However, both mexiletine and lidocaine caused a significant acceleration of slow inactivation with reduced τ_2_ values in a dose-dependent manner, leading to speedy inactivation of either Na_v_1.5 or Na_v_1.7 (*p*<0.001, F = 10.77 for Na_v_1.5; and *p*<0.001, F = 34.16 for Na_v_1.7, one-way ANOVA). Noticeably, mexiletine at 0.3 and 1.0 mM resulted in about 6-fold and 10-fold acceleration of τ_2_ for Na_v_1.5, as compared with about 4-fold and 7-fold change for lidocaine at the same concentration. Similarly, mexiletine also caused about 5- and 9-fold decrease of τ_2_ values for Na_v_1.7, as compared to lidocaine that decreased about 3- and 7-fold of τ_2_ values, indicating stronger effect of mexiletine on slow inactivation of both Na_v_1.5 and Na_v_1.7 (Fig 4 and Table 4). The drugs not only accelerated the slow components but also increased their proportion during the development of closed-state inactivation. The relative weight of slow component increased from 17.2% in the control to 49.6% for 0.3 mM mexiletine and 41.7% for 0.3 mM lidocaine in Na_v_1.5 channel. For Na_v_1.7 channel, the relative weight increased from 26.9% in the control to 58.0% for 0.3 mM mexiletine and 53.3% for 0.3 mM lidocaine. Increasing concentrations of drugs enhanced the slow component further (see Table 4).

**Table 4:** Parameters obtained from fitting closed-state inactivation curves by two-exponential function.

	**Nav1.5**					
	τ**_1_ (ms)**	**Fold change**	**Relative weights**	τ**_2_ (ms)**	**Fold change**	**Relative weights**
**control**	121.9 ± 10.3 (n = 22)		82.8%	2188.1 ± 417.7		17.2%
**mexiletine 0.3 mM**	87.4 ± 13.6 (n = 13)	-1.4	50.4%	385.0 ± 50.6***	-5.7	49.6%
**mexiletine 1.0 mM**	64.9 ± 8.9 (n = 11)**	-1.9	43.2%	212.5 ± 27.6***	-10.3	56.8%
**lidocaine 0.3 mM**	108.8 ± 21.4 (n = 13)	-1.1	58.3%	627.4 ± 195.1***	-3.5	41.7%
**lidocaine 1.0 mM**	107.7 ± 13.2 (n = 15)	-1.1	47.5%	301.7 ± 40.0***	-7.3	52.5%
	**Na_v_1.7**					
	τ**_1_ (ms)**	**Fold change**	**Relative weights**	τ**_2_ (ms)**	**Fold change**	**Relative weights**
**control**	126.7 ± 16.8 (n = 10)		73.1%	2370.2 ± 376.8		26.9%
**mexiletine 0.3 mM**	90.4 ± 5.3 (n = 15)	-1.4	42.0%	489.7 ± 38.9***	-4.8	58.0%
**mexiletine 1.0 mM**	74.4 ± 7.7 (n = 21)**	-1.7	34.5%	270.4 ± 13.3***	-8.8	65.5%
**lidocaine 0.3 mM**	114.8 ± 14.2 (n = 16)	-1.1	46.7%	791.2 ± 137.3***	-3.0	53.3%
**lidocaine 1.0 mM**	104.0 ± 12.3 (n = 10)	-1.2	38.4%	342.2 ± 47.9***	-6.9	61.6%

Note: Asterisk indicates values derived from fitting two-exponential function that differs from the corresponding values in control (drug-free) condition.

**p < 0.01 and ***p < 0.001, one-way ANOVA.

Error estimates on relative weights were not obtained due to the values calculated from averaged data.

### Prolonged recovery from inactivation by mexiletine and lidocaine

**Fig 5:**
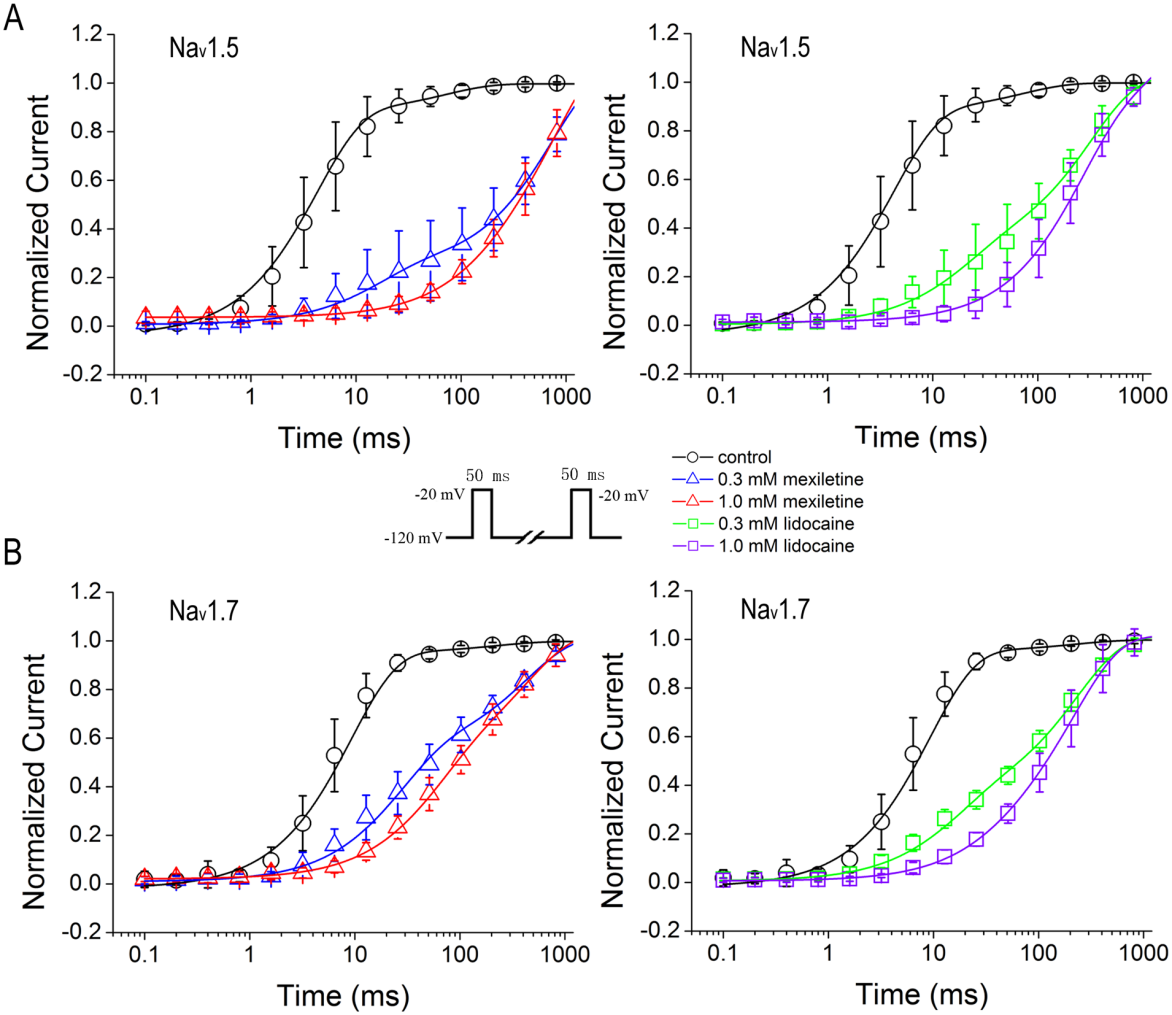
Delayed recovery from inactivation by mexiletine and lidocaine. Recovery of Na_v_1.5 or Na_v_1.7 from inactivation was measured with two-pulse protocol (inset). After 50 ms inactivation pulse to -20 mV, channels were allowed to recover at the holding potential. Normalized peak currents were plotted versus recovery time. The curves were best fitted with two-exponential function. Mexiletine and lidocaine prolonged the recovery from inactivation in Na_v_1.5 (A) and Na_v_1.7 (B) channels. The fast and slow constants as well as their relative weights were summarized in Table 5.

Recovery from inactivation correlates to the use-dependent inhibition that determines the affinity of inactivated sodium channels for drugs. We examined the process for recovery from inactivation resulted from block of mexiletine and lidocaine by utilizing a standard two-pulse protocol consisting of a depolarizing pulse to -20 mV from -120 mV lasting 50 ms for inactivating channels, and a variable duration step at -120 mV for recovery. The availability of channels after the end of recovery interval was assessed with a standard test pulse at -20 mV for 50 ms, and normalized currents were plotted against the recovery interval (Fig 5, inset). We took two-exponential function to fit the recovery from inactivation curves that show the time-dependent recovery constants of Na_v_1.5 and Na_v_1.7 in the presence or absence of mexiletine and lidocaine (Fig 5A and 5B). In control condition, the fast constants (τ_1_) of both channels are in the same range with Na_v_1.5 τ_1 control_ at 5.3 ± 0.5 ms (n = 20) and Na_v_1.7 τ_1 control_ at 3.6 ± 0.2 ms (n = 23) (*p* = 0.0546, unpaired t-test), whereas the slow time constant of Na_v_1.5 (τ_2 control_ = 132.9 ± 27.9 ms) is almost doubled, as compared to Na_v_1.7 (τ_2control_ = 70.1 ± 27.0 ms) (*p*<0.05, unpaired t-test).

Mexiletine at 0.3 mM and 1 mM significantly prolonged the fast recovery time constant (τ_1_) of Na_v_1.5 about 4- and 21-fold, respectively; whereas lidocaine at 0.3 mM and 1 mM increased τ_1_ about 2- and 17-fold (Table 5). In contrast, mexiletine at 0.3 mM and 1 mM significantly prolonged the slow recovery time constant (τ_2_) of Na_v_1.5 about 6- and 13-fold, as compared to lidocaine that at both 0.3 mM and 1 mM only increased τ_2_ with about 2-fold (Table 5). For recovery from inactivation of Na_v_1.7, mexiletine had small effects on both fast and slow time constants, as compared to Na_v_1.5. Mexiletine at 0.3 mM and 1 mM increased τ_1_ about 3- and 15-fold, respectively; and τ_2_ about 5-fold and 8-fold, respectively (Table 5). In comparison with mexiletine, lidocaine at 0.3 mM and 1.0 mM increased τ_1_ about 2- and 7-fold, respectively; and τ_2_ only about 3-fold for both concentrations (0.3 and 1.0 mM).

**Table 5:** Parameters obtained from fitting the recovery curves by two-exponential function.

	**Na_v_1.5**					
	τ**_1_ (ms)**	**Fold change**	**Relative weights**	τ**_2_ (ms)**	**Fold change**	**Relative weights**
**control**	5.3 ± 0.5 (n = 20)		84.1%	132.9 ± 27.9		15.9%
**mexiletine 0.3 mM**	21.6 ± 3.3 (n = 9) ***	4.1	19.6%	768.2 ± 119.3***	5.8	80.4%
**mexiletine 1.0 mM**	111.0 ± 44.0 (n = 8) ***	20.9	0.5%	1716.3 ± 880.9***	12.9	99.5%
**lidocaine 0.3 mM**	9.1 ± 0.5 (n = 8) ***	1.7	16.6%	207.0 ± 12.0***	1.6	83.4%
**lidocaine 1.0 mM**	88.1 ± 9.00 (n = 7) ***	16.6	8.7%	297.2 ± 27.9***	2.2	91.3%
	**Na_v_1.7**					
	τ**_1_ (ms)**	**Fold change**	**Relative weights**	τ**_2_ (ms)**	**Fold change**	**Relative weights**
**control**	3.6 ± 0.2 (n = 23)		89.8%	70.1 ± 27.0		10.2%
**mexiletine 0.3 mM**	10.8 ± 1.5 (n = 7) ***	3.0	48.2%	349.1 ± 23.1***	5.0	51.8%
**mexiletine 1.0 mM**	53.6 ± 6.1 (n = 17) ***	14.9	41.1%	551.0 ± 113.1***	7.9	58.9%
**lidocaine 0.3 mM**	6.4 ± 1.9 (n = 6) ***	1.8	27.7%	208.4 ± 23.4***	3.0	72.3%
**lidocaine 1.0 mM**	26.6 ± 9.3 (n = 6) ***	7.4	7.5%	220.4 ± 35.0***	3.1	92.5%

Note: Asterisk indicates values derived from fitting two-exponential function that differs from the corresponding value in control (drug-free) condition with one-way ANOVA.

***p < 0.001

Error estimates on relative weights were not obtained due to the values calculated from averaged data.

The relative weights in slow phase for both Na_v_1.5 and Na_v_1.7 without drugs were about 15.9% and 10.2%, respectively. Raising the drug concentration increased the slow recovering fraction. For example, mexiletine at 0.3 and 1.0 mM increased the weights of Na_v_1.5 in the slow phase to about 80% and 99%, respectively, as compared to 52% and 59% for Na_v_1.7 (Table 5). For lidocaine, it had little differential effects on the relative weights between the two channels although it also increased values to 83.4% and 91.3% at 0.3 and 1.0 mM for Na_v_1.5, respectively, and 72.3% and 92.5% for Na_v_1.7 (Table 5). This suggests that gating properties coupled to slow inactivation might be key determinants of isoform-specific action of anti-arrhythmic drugs.

### Differential use/frequency-dependent inhibition of Na_v_1.5 by mexiletine and lidocaine

**Fig 6:**
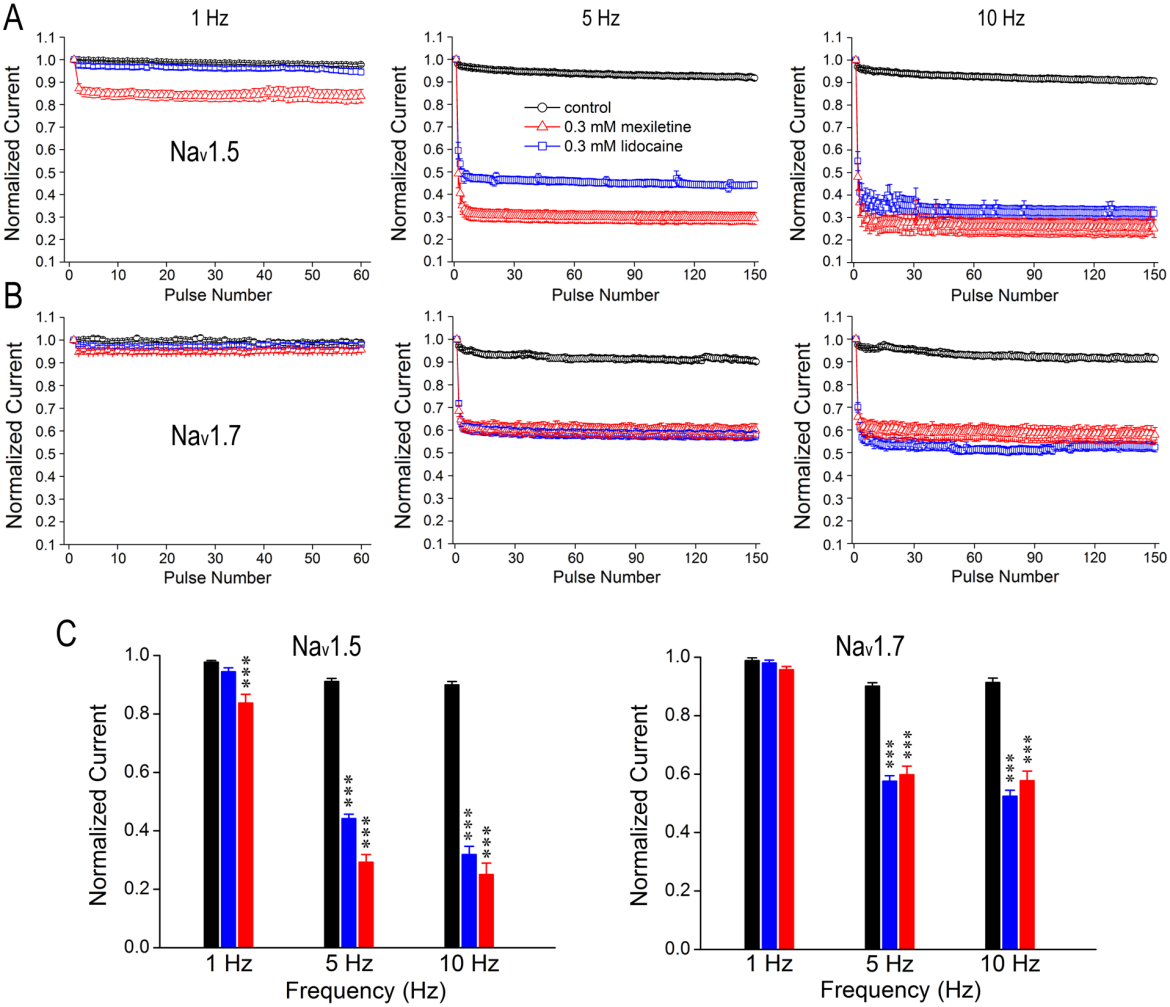
Differential use-dependent inhibition of Na_v_1.5 and Na_v_1.7 by mexiletine and lidocaine. Cells were held at -120 mV and pulsed at -20 mV for three different frequencies (1, 5 and 10 Hz), with interpulse potential set at -120 mV. The peak currents elicited by each pulse were normalized to the current of first pulse and were plotted against the pulse number. Black symbols represent control condition without drugs, while red and blue symbols represent experiments in the presence of 0.3 mM mexiletine or 0.3 mM lidocaine, respectively. A and B, Na_v_1.5 and Na_v_1.7 were stimulated by a train of 60 pulses at 1 Hz and a train of 150 pulses at 5 Hz or 10 Hz in the presence and absence of 0.3 mM mexiletine or lidocaine, respectively. C, Bar graphs represent the relative amplitudes at the last sweep (60^th^ or 150^th^) of use-dependent protocol for each frequency. Increasing the number of pulses resulted in current reduction for Na_v_1.5 in a frequency-dependent manner in the presence of mexiletine or lidocaine at 0.3 mM, whereas mexiletine showed a stronger use-dependent inhibition than lidocaine. In contrast, there is no difference in the inhibition of Na_v_1.7 at 5- and 10-Hz between mexiletine and lidocaine. Besides, the overall current reduction in Na_v_1.5 is more prominent than that of Na_v_1.7 due to the drug effects. Asterisk indicates the significance compared to drug-free (control) condition at each frequency by one-way ANOVA, ***p<0.001.

The significant increase of slow inactivation in the presence of either mexiletine or lidocaine shows that some channels enter into a slow inactivated state during depolarization, thus resulting in accumulation of channels in a drug-modified state and leading to use/frequency-dependent inhibition. To test use-dependent inhibition of Na_v_1.5 or Na_v_1.7 by mexiletine and lidocaine, we applied a series of 60- or 150–50 ms short depolarizing pulses at -20 mV with different frequencies (1 Hz, 5 Hz and 10 Hz). In the absence of drugs, there was no discernable block of channel isoforms when stimulated at 1 Hz and also the currents at 5- and 10-Hz remained above 95% of their initial values (Fig 6A and 6B), indicating that tested channels were able to effectively cycle through the processes of the closed, open and inactivated conformations without obvious reduction in current amplitude.

Following bath application of 0.3 mM mexiletine, the rapid pulsing resulted in a progressive reduction of peak currents of Na_v_1.5, showing the drug-modified use-dependent inhibition of the channel at all frequencies (*p*<0.001, unpaired t-test). However, lidocaine had no inhibition at 1 Hz (*p* = 0.0677, unpaired t-test), and showed less inhibition at both 5- and 10 Hz compared to mexiletine (Fig 6A, *p*<0.001, F = 463.40 for 5 Hz; and *p*<0.001, F = 435.50 for 10 Hz, one-way ANOVA, Bonferroni’s Multiple Comparion Test). This result indicates that Na_v_1.5 is more sensitive to mexiletine in use-dependent manner than lidocaine. In contrast, there is no significant difference in use-dependent inhibition of Na_v_1.7 between mexiletine and lidocaine (*p* = 0.1434 for 1 Hz; *p* = 0.5425 for 5 Hz; and *p* = 0.1845 for 10 Hz, unpaired t-test), although lidocaine had a slightly stronger inhibition at 10 Hz (Fig 6B). It is also noticeable that the overall current reduction of Na_v_1.5 in the presence of either mexiletine or lidocaine is larger than Na_v_1.7 (Fig 6C). In Na_v_1.5 channels, mexiletine caused about 61.89% and 76.03% of reduction at 5 Hz and 10 Hz, respectively; while lidocaine resulted in about 51.51% and 64.58% at 5 Hz and 10 Hz, respectively. In Na_v_1.7 channels, mexiletine-induced current reduction was 33.66% and 34.47% at 5 Hz and 10 Hz, respectively; lidocaine-induced current reduction was about 36.11% and 44.00% at 5 Hz and 10 Hz, respectively.

## Discussion

The objective of the present study was to compare gating properties of cardiac Na_v_1.5 and neuronal Na_v_1.7 channels, and their use-dependent inhibition by two drugs mexiletine and lidocaine that are widely used for cardiac arrhythmia and pain prevention. Due to the lack of specific sodium channel isoform blocker, current clinical therapy often brings side effects for patients who carry *SCN5A* mutations [25] or *SCN*9A mutations [26, 27]. Therefore, detailed characterizations of biophysical properties and their pharmacological responses to mexiletine and lidocaine may encourage the rational design of more potent therapeutically interesting isoform-selective drugs.

In principal, voltage-gated sodium channels undergo the process of resting, open and inactivated states. In this study, voltage-dependent activation of Na_v_1.5 and Na_v_1.7 channels is not affected by mexiletine or lidocaine which is consistent with other reports using *Xenopus* oocytes system [8, 28]. We observed that both of the steady-state fast and slow inactivation curves of either channel isoform were significantly shifted toward more hyperpolarized direction and the shift was correlated with the concentration of drugs. Effects of mexiletine or lidocaine on steady-state slow inactivation are more dramatic by larger leftward shift as compared to fast inactivation. One could assume that drugs bind much more tightly to the slow inactivated state of the channels, which can be explained by the increased fraction of slow phase in closed-state inactivation and recovery. Coexpression with β1 subunit is reported to significantly alter the kinetics and voltage sensitivity of Na_v_1.7 and Na_v_1.8 isoforms expressed in DRG neurons, including the shift of steady-state activation, inactivation as well as the recovery from fast inactivation [4]. In *Xenopus* oocytes co-expressing β1 subunit, lidocaine does not affect the midpoint of activation for Na_v_1.7, but significantly shifts the midpoint activation for Na_v_1.8 to depolarized direction [8], suggesting that the differential modulation is Na_v_1.7 or Na_v_1.8 itself dependent. Although there is a 5.5-mV difference in the steady-state fast inactivation and 24.4-mV difference in steady-state slow inactivation between Na_v_1.5 and Na_v_1.7, the leftward shift in response to mexiletine or lidocaine is similar at low concentration (0.3 mM). This parallel shift resulted from drug-free and drug action implies depolarization-mediated gating processes occurring in isoform-specific block [29] and also an attribution to a combination of lipophilic and increasing amount of voltage-sensor block [13].

Closed-state inactivation can occur at hyperpolarized and modestly depolarized membrane potential. In the present study, we adopted the protocol of inactivating the cell at a slightly depolarized potential of -80 mV. The closed-state inactivation is enhanced by both mexiletine and lidocaine, as seen for accelerated fast and slow time constants of both τ_1_ and τ_2_ values. In comparison with lidocaine, mexiletine shows a stronger effect on speeding slow component of closed-state inactivation and relative weights contributed by slow component of closed-state inactivation for both Na_v_1.5 and Na_v_1.7. This could in part explain a larger reduction of current resulted from use-dependent inhibition of Na_v_1.5 channels by mexiletine, which is consistent with the report that lidocaine interacts with rat Na_v_1.4 channels by enhancing transition to slow inactivated state [30–32].

Recovery from inactivation is a direct way to test the affinity of inactivated channels to anti-arrhythmic drugs. In general, the kinetics of Na_v_1.7 is faster than Na_v_1.5 in the absence or presence of drugs. Mexiletine resulted in fast and slow recovery constants of Na_v_1.5 more than two times longer of Na_v_1.7 (Table 5). The fold changes induced by mexiletine in both Na_v_1.5 and Na_v_1.7 were also more prominent compared to the same concentration of lidocaine. Clinical studies reveal a comparison between intravenous mexiletine and lidocaine for the treatment of ventricular arrhythmias by showing that mexiletine results in greater suppression of ventricular premature depolarization [33]. This is consistent with our finding that mexiletine had a strong impact on recovery from inactivation of Na_v_1.5 channel than lidocaine. The isoform-specific block might result from differences in gating rather than receptor differences. This is supported by experiments that recovery from drug block is relatively insensitive to isoform differences in fast inactivation gating which indicates that other gating differences might affect isoform-specific drug action [29]

Anti-arrhythmic drugs are known to block sodium currents more potently during repetitive depolarization (use-dependent inhibition) than during infrequent stimuli from rest (tonic block) [34]. Three mechanisms have been shown for blocking effects of anti-arrhythmic drugs on Na^+^ channels: 1, block of open or inactivated state; 2, block of closed state; and 3, fast-flicker block of the open channel [13]. Considering the use-dependent inhibition, only the first one has a high enough affinity to be potentially useful as an anti-arrhythmic drug. The anti-arrhythmic drug receptor in sodium channels is formed by residue from multiple S6 segments and seems highly conserved among Na^+^ channel isoforms [35]. In our experiments both mexiletine and lidocaine produced more use-dependent inhibition of peak Na_v_1.5 current during trains of short depolarization than that of Na_v_1.7 current. This phenomenon becomes more prominent at faster stimulation rates. One would expect a larger amount of use-dependent block when prolonging the depolarization [36]. Based on the data obtained from steady-state slow inactivation, closed-state inactivation and recovery from inactivation, we propose that the tight binding of drugs to slow component of inactivation might be a key criterion to determine the use-dependent effect of drugs. In addition, other reasons also might explain part of the effects. First, cardiac sodium channels have an intrinsically higher binding affinity for lidocaine like drugs. Second, anti-arrhythmics preferentially target the inactivated state of sodium channels. Cardiac sodium channels spend a large fraction of time in this high-affinity inactivated state because the inactivated state in cardiac action potential is relatively longer compared to neuronal channels [37]. It has also been reported that other sodium channel blocker ranolazine, an antianginal with promise as an antiarrhythmic drug, can induce similar use-dependent phenotype in various sodium channel isoforms, with larger steady-state block in Na_v_1.7 than Na_v_1.5 isoform [19, 38].

Our observations show that structure-related anti-arrhythmic drugs have different use-dependent effects on the same sodium channel isoform with mexiletine exerting stronger effects on Na_v_1.5 channel than lidocaine. We propose that the differences in potency are most likely attributed to the change of chemical composition in the aliphatic chain between them. Both mexiletine and lidocaine possess an ionizable amino group and an aromatic ring which are recognized as pharmacophore moieties of sodium channel blocking to match the aromatic amino acids of the receptor, involving specific π-cation and hydrophobic interactions, respectively [39–42]. However, even substitutions at the asymmetric center of mexiletine can greatly enhance the drug potency and minimize or change the effects caused by the structural modifications at the aromatic ring. Therefore, the difference on the asymmetric carbon atom between mexiletine and lidocaine might be pivotal for their potency [40].

It is well accepted that the four S6 segments formed the inner vestibule of the sodium channel pore, which contains the high-affinity use-dependent binding site for local anesthetics (LAs) and anti-arrhythmic drugs. Drug-binding affinity to gating state of sodium channel plays a major role in drug-target interaction [43]. In Na_v_1.5 channel, the local binding site of the blocker is represented by a cluster of residues in the DIII and DIV S6 segments, including DIVS6-Phe1759, DIVS6-Tyr1766 and DIIIS6-Leu1461 through mutational studies [35, 43, 44]. In general, the mutational effects on LA block across three sodium channel isoforms, Na_v_1.2, Na_v_1.4 and Na_v_1.5 have been reported to be similar or identical. The gating properties of certain sodium channels are the major determinants for the binding of sodium channel blockers as well as access to the binding site, which in turn affects their clinical efficacy [45]. However, the structural changes at the selectivity filter region might also be the crucial determinants for the pharmacological response to sodium channel blockers [45, 46]. Therefore, further systematic studies will be required to test the role of different residues for the different modulation of Na_v_1.5 and Na_v_1.7 by anti-arrhythmics. For example, the S4-S5 linker in domain IV, highly conserved among sodium channel isoforms, has been found in Na_v_1.5 with cardiac disorders and Na_v_1.7 with paroxysmal extreme pain disorders [43, 47]. These disease-related mutations have been characterized to affect the efficacy of anti-arrhythmics to inhibit the sodium conductance [21].

In conclusion, we show that Na_v_1.5 and Na_v_1.7 channels exhibit altered electrophysiological properties in the absence or presence of mexiletine and lidocaine. The steady-state fast and slow inactivation shift in the hyperpolarized direction due to the drug effects. Mexiletine exerts more inhibition on the closed-state inactivation and recovery from inactivation in both channels compared to lidocaine. Furthermore, mexiletine causes more use-dependent inhibition on Na_v_1.5 than Na_v_1.7, which can be attributed to tight binding of drug to slow inactivation. As effective anti-arrhythmic drugs, mexiletine and lidocaine share common characteristics such as high affinity and use-dependent block of Na^+^ current at high heartbeat. Although different sodium channels have some similar functional characteristics, there are differences in gating properties to distinguish the two isoforms that likely contribute to their pharmacology. Understanding the gating profiles in response to mexiletine and lidocaine may help explain or predict the drug effectiveness for ventricular tachycardia as well as advance in new designs of safe and specific sodium channel blockers pain with few side effects.
